# Serum Calcium Levels Are Associated with Novel Cardiometabolic Risk Factors in the Population-Based CoLaus Study

**DOI:** 10.1371/journal.pone.0018865

**Published:** 2011-04-21

**Authors:** Idris Guessous, Olivier Bonny, Fred Paccaud, Vincent Mooser, Gérard Waeber, Peter Vollenweider, Murielle Bochud

**Affiliations:** 1 Community Prevention Unit, Institute of Social and Preventive Medicine, Centre Hospitalier Universitaire Vaudois, University of Lausanne, Lausanne, Switzerland; 2 Unit of Population Epidemiology, Division of Primary Care Medicine, Department of Community Medicine, Primary Care and Emergency Medicine, Geneva University Hospitals, Geneva, Switzerland; 3 Service of Nephrology and Hypertension, Lausanne University Hospital and Department of Pharmacology and Toxicology, University of Lausanne, Lausanne, Switzerland; 4 Genetics Division, GlaxoSmithKline Research and Development, King of Prussia, Pennsylvania, United States of America; 5 Department of Medicine, Internal Medicine, Centre Hospitalier Universitaire Vaudois, Lausanne, Switzerland; Innsbruck Medical University, Austria

## Abstract

**Background:**

Associations of serum calcium levels with the metabolic syndrome and other novel cardio-metabolic risk factors not classically included in the metabolic syndrome, such as those involved in oxidative stress, are largely unexplored. We analyzed the association of albumin-corrected serum calcium levels with conventional and non-conventional cardio-metabolic risk factors in a general adult population.

**Methodology/Principal Findings:**

The CoLaus study is a population-based study including Caucasians from Lausanne, Switzerland. The metabolic syndrome was defined using the Adult Treatment Panel III criteria. Non-conventional cardio-metabolic risk factors considered included: fat mass, leptin, LDL particle size, apolipoprotein B, fasting insulin, adiponectin, ultrasensitive CRP, serum uric acid, homocysteine, and gamma-glutamyltransferase. We used adjusted standardized multivariable regression to compare the association of each cardio-metabolic risk factor with albumin-corrected serum calcium. We assessed associations of albumin-corrected serum calcium with the cumulative number of non-conventional cardio-metabolic risk factors.

We analyzed 4,231 subjects aged 35 to 75 years. Corrected serum calcium increased with both the number of the metabolic syndrome components and the number of non-conventional cardio-metabolic risk factors, independently of the metabolic syndrome and BMI. Among conventional and non-conventional cardio-metabolic risk factors, the strongest positive associations were found for factors related to oxidative stress (uric acid, homocysteine and gamma-glutamyltransferase). Adiponectin had the strongest negative association with corrected serum calcium.

**Conclusions/Significance:**

Serum calcium was associated with the metabolic syndrome and with non-conventional cardio-metabolic risk factors independently of the metabolic syndrome. Associations with uric acid, homocysteine and gamma-glutamyltransferase were the strongest. These novel findings suggest that serum calcium levels may be associated with cardiovascular risk via oxidative stress.

## Introduction

Increased serum calcium concentration has been associated with high blood pressure (BP)[1], impaired glucose tolerance [2], and dyslipidemia [3], [4]. More recently, increased serum calcium concentration has been described as a feature of the metabolic syndrome (MSy) [5].

Cardio-metabolic components not classically included in the MSy have been associated with the risk of cardiovascular disease and include components related to adiposity[6], blood lipids [7], and insulin resistance [8]. In addition, factors related to inflammation and oxidative stress have been recently associated with the MSy. For example, observations suggest that serum levels of uric acid, homocysteine or gamma-glutamyltransferase (GGT) might be linked to the MSy [9]–[11].

While associations of serum calcium with the conventional components of the MSy on one hand [5], and of non-conventional cardio-metabolic (NCCM) risk factors with the MSy on the other hand have been explored [12]–[18], the relationship between serum calcium and NCCM risk factors has not been characterized in the general population. We analyzed the association of albumin-corrected serum calcium (Ca_c_) with conventional and a broad range of NCCM risk factors in the Swiss population-based CoLaus study.

## Methods

### Objectives

The objectives were to analyze the association of albumin-corrected serum calcium (Ca_c_) with conventional and non-conventional cardio-metabolic (NCCM) risk factors in a general adult population.

### Participants

The sampling procedure of the CoLaus study has been described elsewhere [19]. Briefly, a simple, non-stratified random sample of 35% of the overall population was drawn. The following inclusion criteria applied: a) written informed consent; b) aged 35–75 years; c) willingness to take part in the examination and donate blood sample; d) Caucasian origin.

### Assessment process and clinical data

Recruitment began in June 2003 and ended in May 2006. All participants attended the outpatient clinic of the University Hospital of Lausanne in the morning after an overnight fast. Data were collected by trained field interviewers using standardized questionnaires. Body mass index (BMI) was defined as weight/height^2^. Waist and hip circumferences were measured as recommended [20]. Fat and fat-free masses were assessed by electrical bioimpedance [21] using the Bodystat® 1500 analyzer (Isle of Man, British Isles). BP was measured thrice on the left arm after at least 10 minutes rest in the seated position using a clinically validated automated oscillometric device (Omron® HEM-907, Matsusaka, Japan) with a standard cuff, or a large cuff if arm circumference was ≥33 cm. The average of the last two BP readings was used for analyses. Hypertension was defined as mean systolic BP (SBP)≥140 mmHg or mean diastolic BP (DBP)≥90 mmHg or presence of anti-hypertensive medication. For the purpose of the present analysis, smoking was defined as present if a participant reported to be a current smoker at the time of examination, regular alcohol consumption was defined as present for participants reporting to drink alcohol at least once a day, and post-menopausal status was self-reported. Diabetes was defined as a fasting glucose≥7 mmol/L and/or presence of antidiabetic drug treatment (insulin or oral drugs).

### Biologic data

The complete description of markers and types of assay used for the purpose of this analysis is reported in **Table S1**. Total serum calcium was measured by O-cresolphtalein and albumin by bromocresol green. Ca_c_ was calculated using the following formula: Ca_c_ = Serum total calcium-0.012 (serum albumin/0.9677−39.55). This formula was derived by the central laboratory of the University Hospital of Lausanne (Centre Hospitalier Universitaire Vaudois, CHUV) based on data from 320 consecutive outpatients without disorders of phosphocalcic metabolism. In CoLaus, Cac using this formula presented no residual correlation with serum albumin. Glomerular filtration rate was estimated using the abbreviated Modification of the Diet in Renal Disease (MDRD) formula: 186×(serum creatinine [µmol/L]/88.4)^(−1.154)^×age^(−0.203)^×F, where F = 1 for men and F = 0.742 for women [22].

### Conventional and non-conventional cardio-metabolic components

The MSy was defined using the Adult Treatment Panel III criteria [23] as presenting with 3 or more of the following criteria: (1) waist circumference≥102 cm in men and ≥88 cm in women; (2) SBP≥130 mmHg or DBP≥85 mmHg or medication use; (3) triglycerides≥1.69 mmol/L (150 mg/dL); (4) HDL cholesterol<1.04 mmol/L (40 mg/dL) in men and<1.30 mmol/L (50 mg/dL) in women, or medication use; (5) fasting plasma glucose≥6.1 mmol/L (110 mg/dL) or medication use. We did not consider drug treatment for elevated triglycerides in the definition of elevated triglycerides. Components of the MSy were defined as *conventional* cardio-metabolic risk factors. Cardio-metabolic components that are not included in the MSy were defined as *non-conventional* cardio-metabolic (NCCM) risk factors and classified into the following four groups: (i)*Adiposity*: fat mass (kg), leptin (ng/mL); (ii)*Blood lipids*: LDL-cholesterol (mmol/L), LDL size (angström), apolipoprotein B (mg/dL); (iii)*Insulin resistance*: fasting insulin (µU/mL), adiponectin (µg/mL); (iv)*Inflammation-oxidative stress*: ultrasensitive CRP (mg/L), serum uric acid (µmol/L), homocysteine (µmol/L), GGT (UI/L).

### Ethics

The CoLaus study complied with the Declaration of Helsinki and was approved by the local Institutional Ethic Committee (Baudraz Marcel-André, Boillat Marcel-André, Gardaz Jean-Patrice, Joye Charles, Lejeune Ferdy, Pannatier André, Reymond Catherine, Rossel Martine, Burnand Bernard). All participants gave written informed consent.

### Statistical analyses

Statistical analyses were performed using Stata 10.0 (Stata Corp, College Station, USA). Continuous variables were expressed as mean ± standard deviation (s.d.) or as median and interquartile range [IQR]. Categorical variables were expressed as number of subjects and percentage.

Linear and median regressions were used to assess the association of serum Ca_c_ with conventional and NCCM risk factors. Non-normally distributed variables were transformed (log or cubic transformation). Associations were considered with serum Ca_c_ taken as quintiles (quintiles analysis) and as a continuous variable (standardized and score analyses).

In quintiles analysis, interaction with gender and trends by quintiles were assessed using median regression, adjusting for age, smoking, alcohol consumption, menopausal status, eGFR, and thiazide use. Robust regression was used whenever models were unstable. Adjusted standardized multivariable linear regression was used to compare the association with Ca_c_ of each of the cardio-metabolic risk factors. S.d. equals 1 for all standardized beta coefficients. Associations of serum Ca_c_ with the number of cardio-metabolic risk factors were then assessed. Each conventional cardio-metabolic risk factor was included in a score using the ATPIII classification (MSy score; ranged from 0 to 5). For the NCCM score, subjects in the upper tertile of the risk factor were assigned a value of 1, whereas subjects in the first and second tertiles were assigned a value of 0 for each NCCM risk factor positively correlated with cardiovascular disease, and vice versa for NCCM risk factor negatively correlated with cardiovascular disease. Positive and negative correlations were based on the existing literature. Only NCCM risk factors that showed a statistically significantly trend with Ca_c_ in quintiles analyses were entered into the NCCM score. The linearity of association between serum Ca_c_ and MSy score, or between serum Ca_c_ and NCCM score, were tested using median regression. When linearity failed to be rejected, linear trends by score were tested. NCCM score analysis was further adjusted for conventional cardio-metabolic risk factors. The association analyses between Ca_c_ and the two scores were performed with and without adjustment for BMI. We only included in the analysis subjects for whom all variables of interest for the purpose of this study were available. We conducted sensitivity analyses using an alternative formula to correct serum calcium for albumin as proposed by Ahlström et al, i.e. calcium+0.019* (42-albumin) [5]. We also performed sensitivity analyses by including all CoLaus participants in the analyses, which increases the sample size.

## Results

All variables of interest were not available in 1’957 participants, thus a total of 4,231 subjects were included in the analysis. This represents 68.4% (4,231/6,188) of the entire CoLaus sample. Subjects included in the analysis differed from excluded participants in several aspects (**Table S2**).

Clinical characteristics by gender are detailed in **Table 1**. Men had a higher mean BMI than women, and a higher prevalence of cigarette smoking, and of regular alcohol consumption, than women. Men were also more likely to have hypertension and diabetes than women. Mean serum calcium was similar between genders whereas Ca_c_ was slightly lower in men than in women (2.21±0.08 vs. 2.22±0.09 mmol/L).

**Table 1:** Characteristics of the 4,231 individuals from the CoLaus study.

	Men (N = 1,976)		Women (N = 2,255)		
	Mean or %	SD	Mean or %	SD	P value
Age (years)	53.5	10.9	53.8	10.8	0.466
Body mass index (kg/m^2^)	27.1	3.8	25.2	4.4	**<0.001**
Cigarette smoking (%)	27.2	-	23.8	-	**0.010**
Regular alcohol consumption (%)	35.6	-	15.6	-	**<0.001**
Post-menopause (%)	-	-	57.0	-	
Fasting blood glucose≥7 mmol/L (%)	10.2	-	3.46	-	**<0.001**
Fasting blood glucose≥6.1 mmol/L* (%)	20.7		7.8		**<0.001**
SBP≥140 and or DBP≥90 mmHg (%)	45.6	-	31.4	-	**<0.001**
SBP≥130 and or DBP≥80 mmHg (%)*	62.5	-	45.7	-	**<0.001**
Waist circumference≥102 cm in men or≥88 cm in women* (%)	27.5		31.6		**0.004**
HDL-cholesterol<1.04 in men or<1.30 mmol/L in women* (%)	11.3		13.5		**0.028**
Triglycerides≥1.69 mmol/L* (%)	35.2		16.0		**<0.001**
ATPIII Metabolic syndrome (%)	22.8	-	13.7		**<0.001**
eGFR (ml/min/1.73 m^2^)	86.0	16.9	80.6	15.3	**<0.001**
Serum calcium (mmol/L)	2.29	0.09	2.29	0.10	0.162
Serum albumin (g/L)	44.6	2.5	43.8	2.4	**<0.001**
Albumin-corrected calcium (mmol/L)	2.21	0.08	2.22	0.09	**0.017**

*criteria used for the ATPIII Metabolic syndrome definition. SBP, systolic blood pressure; DBP, diastolic blood pressure; HDL-cholesterol, high-density-lipoprotein cholesterol; eGFR, GFR estimated using the MDRD formula.

The distributions of conventional and NCCM risk factors are presented in **Table 2**. With the exception of HDL-cholesterol, the means/medians of all conventional cardio-metabolic risk factors were higher in men than in women. The MSy was present in 451 men (23%) and 310 women (14%).

**Table 2:** Values of conventional and nonconventional parameters involved in the metabolic syndrome in the 4,231 individuals from the CoLaus study.

	Men (N = 1,976)		Women (N = 2,255)		
	Mean or %	SD	Mean or %	SD	P value
**Conventional risk factors**					
SBP (mmHg)	133	17	125	18	**<0.001**
DBP (mmHg)	81.8	10.5	77.8	10.6	**<0.001**
HDL-cholesterol (mmol/L)	1.41	0.34	1.79	0.42	**<0.001**
Triglycerides (mmol/L)*	1.3	0.9–2.0	1.0	0.8–1.4	**<0.001**
Fasting blood glucose (mmol/L)	5.83	1.20	5.34	0.89	**<0.001**
Waist circumference (cm)	97.4	10.5	83.7	11.6	**<0.001**
**Adiposity**					
Fat mass (kg)	20.7	7.3	23.4	8.5	**<0.001**
Leptin (ng/mL)*	6.42	4.0–11.1	14.8	8.8–24.0	**<0.001**
**Blood lipids**					
LDL-cholesterol (mmol/L)	3.46	0.91	3.30	0.93	**<0.001**
LDL size (angstrom)*	272	268–274	274	271–275	**<0.001**
Apolipoprotein B (mg/dL)*	152	109–222	135	97–199	**<0.001**
**Insulin resistance**					
Fasting insulin (µU/mL)*	8.00	5.8–12.0	6.29	4.6–9.5	**<0.001**
Adiponectin (µg/mL)*	6149	4041–9124	10289	6696–15184	**<0.001**
**Inflammation**					
CRP (mg/L)*	1.3	0.7–2.6	1.4	0.7–3.0	**0.427**
**Oxidative stress**					
Serum uric acid (µmol/L)	10.4	8.8–12.7	8.8	7.3–10.7	**<0.001**
Homocysteine (µmol/L)*	29.5	21.0–46.5	16.0	12.0–24.0	**<0.001**
GGT (UI/L)*	365	76	271	67	**<0.001**

See footnote of Table 1. LDL-cholesterol, low-density-lipoprotein cholesterol, GGT, gamma-glutamyltransferase. * Data are median, interquartile range, and 2-sample Wilcoxon rank-sum test; uCRP = ultrasensitive C reactive protein.

As to NCCM risk factors, women had higher median fat mass and leptin levels than men. LDL-cholesterol and apolipoprotein B were higher in men than in women, whereas LDL median size was lower in men. Compared to women, levels of serum uric acid, homocysteine and GGT were higher in men.

**Table 3** shows adjusted associations of conventional and NCCM risk factors with Ca_c_ quintiles. Among the conventional risk factors, SBP, DBP, triglycerides and fasting blood glucose were all significantly higher across increasing quintiles of Ca_c_.

**Table 3:** Adjusted conventional and non-conventional metabolic components, by sex-specific albumin-corrected calcium quintiles. (Men+Women, N = 4,231).*

	Q1[2.11 mmol/L](N = 903)	Q2[2.17 mmol/L](N = 800)	Q3[2.22 mmol/L](N = 946)	Q4[2.26 mmol/L](N = 766)	Q5[2.33 mmol/L](N = 816)	P value for trend
**CONVENTIONAL**						
SBP (mm Hg)	125	127	128	129	130	**0.000**
	[124–126]	[126–127]	[127–128]	[128–130]	[129–131]	
DBP (mm Hg)	77.5	78.5	79.0	79.8	80.2	**0.000**
	[76.9–78.1]	[78.1–78.9]	[78.8–79.4]	[79.4–80.2]	[79.7–80.9]	
HDL-cholesterol (mmol/L)**	1.60	1.57	1.59	1.58	1.60	0.32
	[1.58–1.62]	[1.56–1.58]	[1.57–1.59]	[1.56–1.59]	[1.58–1.62]	
Triglycerides (mmol/L)	1.08	1.15	1.17	1.21	1.23	**0.000**
	[1.05–1.12]	[1.12–1.17]	[1.15–1.19]	[1.19–1.24]	[1.20–1.27]	
Fasting blood glucose (mmol/L)	5.36	5.40	5.41	5.44	5.44	**0.01**
	[5.32–5.39]	[5.38–5.43]	[5.39–5.43]	[5.41–5.46]	[5.40–5.48]	
Waist circumference (cm)	87.6	89.1	89.1	89.7	89.3	0.07
	[86.8–88.3]	[88.6–89.7]	[89.7–89.5]	[89.1–90.2]	[88.5–90.1]	
**NON CONVENTIONAL**						
ADIPOSITY						
Fat mass (kg)	20.5	20.6	20.9	21.2	21.5	**0.001**
	[20.1–20.9]	[20.3–20.9]	[20.7–21.2]	[20.9–21.5]	[21.1–21.9]	
Leptin (ng/mL)	11.6	10.9	11.0	10.8	11.0	0.41
	[11.2–12.0]	[10.7–11.3]	[10.8–11.3]	[10.5–11.1]	[10.6–11.5]	
LIPID						
LDL-cholesterol(mmol/L)	3.12	3.25	3.33	3.43	3.50	**<0.001**
	[3.08–3.16]	[3.22–3.28]	[3.30–3.35]	[3.40–3.46]	[3.45–3.55]	
LDL size (angström)	273.0	272.7	272.6	272.5	272.4	**0.001**
	[272.8–273.2]	[272.6–272.9]	[272.5–272.8]	[272.3–272.6]	[272.2–272.6]	
Apolipoprotein B (mg/dL)	141	143	143	145	144	0.58
	[136–146]	[140–147]	[141–146]	[141–148]	[139–149]	
INSULIN						
Fasting insulin (μU/mL)	6.62	7.03	7.27	7.58	7.78	**<0.001**
	[6.39–6.84]	[6.87–7.19]	[7.14–7.40]	[7.42–7.74]	[7.54–8.01]	
Adiponectin (µg/mL)	9064	8544	8370	8058	7957	**<0.001**
	[8739–9389]	[8315–8774]	[8184–8557]	[7823–8293]	[7619–8296]	
INFLAMMATION						
uCRP (mg/L)	1.37	1.38	1.40	1.41	1.42	0.51
	[1.27–1.47]	[1.31–1.45]	[1.34–1.45]	[1.34–1.48]	[1.32–1.53]	
OXIDATIVE STRESS						
Serum uric acid (µmol/L)	292	307	311	320	322	**<0.001**
	[289–296]	[305–310]	[309–313]	[317–322]	[318–325]	
Homocysteine (µmol/L)	9.12	9.53	9.74	10.03	10.19	**<0.001**
	[8.99–9.25]	[9.44–9.62]	[9.66–9.81]	[9.93–10.12]	[10.05–10.33]	
GGT (UI/L)	20.45	22.42	23.23	24.50	25.07	**<0.001**
	[19.89–21.01]	[22.03–22.81]	[22.91–23.55]	[24.10–24.91]	[24.49–25.65]	

SBP = systolic blood pressure; DBP = diastolic blood pressure; uCRP = ultrasensitive C reactive protein.

*Results are medians [95% confidence intervals] adjusted for age, smoking, alcohol consumption, menopause status, eGFR, and thiazide use.

**Robust regression was used to model HDL-cholesterol.

Among NCCM risk factors, fat mass increased with increasing quintiles of Ca_c_ (p value = 0.001); LDL-cholesterol increased (p value<0.001) whereas LDL size decreased as Ca_c_ quintiles rose (p value = 0.001). Factors related to insulin resistance were significantly associated with quintiles of Ca_c_: fasting insulin was positively and adiponectin negatively associated with Ca_c_ quintiles. Ultrasensitive CRP levels rose with Ca_c_ quintiles, thought not significantly. Among NCCM risk factors related to oxidative stress, all were increasing with increasing quintiles of Ca_c_ (all with p<0.01). Gender interaction was only statistically significant for GGT (p<0.001) (sex-specific associations between cardio-metabolic risk factors and Ca_c_ quintiles are provided in **Tables S3 and S4**). Sensitivity analyses including all CoLaus participants led to similar results, except for a significant association of CRP with calcium quintiles in men. As CRP was not significantly associated with calcium quintiles in participants who actually contribute to the final statistical models, we did not include CRP in the score.

The eight NCCM associated with Ca_c_ in the adjusted quintiles analyses (1) fat mass, (2) LDL-cholesterol, (3) LDL size, (4) insulin, (5) adiponectin, (6) uric acid, (7) homocysteine, and (8) GGT) were included in further analyses. The largest pairwise Spearman rank correlations were between SBP and DBP (0.79), and between fat mass and waist circumference (0.57).

**Figure 1:**
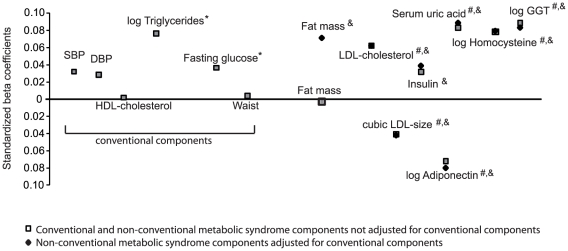
Adjusted standardized associations between metabolic components and albumin-corrected calcium. Only non-conventional metabolic syndrome components that were associated with albumin-corrected calcium in adjusted quintiles analyses: fat mass, LDL-chol, LDL size, insulin, adiponectin, uric acid, homocysteine, and GGT). Age, sex, smoking, alcohol, menopausal status, eGFR and thiazide use were used as covariates in the models. * = P value<0.05 for adjusted conventional cardio-metabolic risk factors. # = P value<0.05 for adjusted non-conventional cardio-metabolic risk factors (not adjusted for conventional factors). & = P value<0.05 for adjusted non-conventional metabolic syndrome components (adjusted for conventional components).

**Figure 1** illustrates adjusted standardized associations between cardio-metabolic risk factors and Ca_c_ taken as the continuous dependent variable. All conventional cardio-metabolic risk factors were positively associated with Ca_c_. Triglycerides was the conventional cardio-metabolic risk factor most strongly associated with Ca_c_ (β = +0.076). NCCM risk factors related to oxidative stress were the factors most strongly and positively associated with Ca_c_. Adjusted for age, sex, smoking, alcohol, menopausal status, eGFR, and thiazide use, standardized beta coefficients for acid uric, logGGT and logHomocysteine were +0.083, +0.089, and +0.078, respectively. Adiponectin had the most important negative association with Ca_c_ (β = −0.072). Except for fat mass, further adjustment for conventional cardio-metabolic risk factors did not meaningfully change the magnitude of the association between NCCM risk factors and Ca_c_. The standardized beta coefficient of fat mass increased from −0.002 to 0.071 after further adjustment for conventional cardio-metabolic risk factors. Further analyses showed that fat mass was mostly influenced by the introduction of waist circumference in the model. Standardized beta coefficients are presented by gender in **Table S5.**

**Figure 2:**
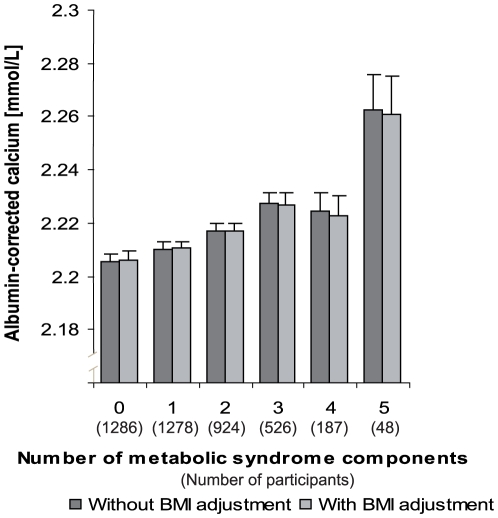
Albumin-corrected calcium, by number of metabolic syndrome components: adjusted for age, sex, smoking, alcohol, menopause status, eGFR, and thiazide use. P value for trend <0.001 for model with and without BMI adjustment.

Respectively 1286 (30.4%), 1278 (30.2%), 924 (21.8%), 526 (12.4%), 187 (4.4%) and 48 (1.1%) subjects had 0, 1, 2, 3, 4, or 5 conventional cardio-metabolic risk factors (MSy score). **Figure 2** illustrates the association between MSy score and Ca_c_. Ca_c_ increased with the number of conventional cardio-metabolic risk factors (p for linear trend<0.001). Further adjustment for BMI did not change the magnitude or the significance of the association. Linear trends were significant for men and women considered separately (**Table S6**). Sensitivity analyses using the alternate formula (calcium + 0.019 * [42−albumin]) [5] showed higher corrected calcium levels for each category of MSy or NCCM components, but similar trends and levels of statistical significance were observed, which leads the same conclusions (data available upon request).

**Figure 3:**
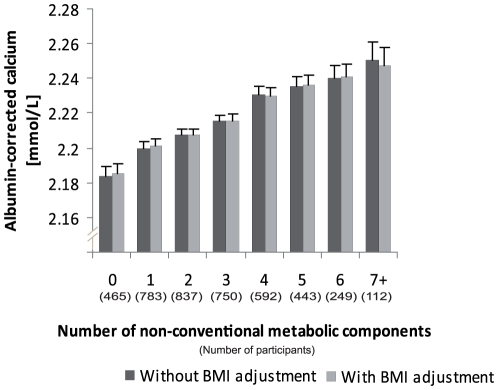
Albumin-corrected calcium, by number of non-conventional metabolic syndrome components: adjusted for age, sex, smoking, alcohol, menopause status, eGFR, thiazide use, and conventional metabolic syndrome components. P value for trend<0.001 for model with and without BMI adjustment. Only non-conventional metabolic syndrome components that were associated with albumin-corrected calcium in adjusted quintiles analyses (fat mass, LDL-chol, LDL size, insulin, adiponectin, uric acid, homocysteine, and GGT) are taken into account.

Because only 16 (0.37%) subjects presented all 8 NCCM risk factors, categories 7 and 8 were pooled. Thus, respectively 465 (11.0%), 783 (18.5%), 837 (19.8%), 750 (17.7%), 592 (14.0%), 443 (10.5%), 249 (5.9%), and 112 (2.6%) had 0, 1, 2, 3, 4, 5, 6, or 7+ NCCM risk factors (NCCM score). The mean number of NCCM per subjects was 2.7 (SD = 1.83). **Figure 3** illustrates the association between NCCM score and Ca_c._ Ca_c_ increased with the number of NCCM risk factors (p for linear trend<0.001). Further adjustment for BMI did not change the magnitude and significance of the association. Linear trends were significant for men and women and considered separately (**Table S7**).

## Discussion

We found serum calcium to be strongly associated with conventional MSy components, in particular fasting serum glucose and serum triglycerides. Serum calcium showed a positive trend with the number of MSy components, independently of BMI. Ca_c_ increased from 0 to 3 components, followed by a plateau and a final rise. The association of serum calcium with the number of MSy components was recently reported in a population-based study of 1000 elderly subjects [5]. Our study confirmed this association in a larger population of subjects aged 35–75 years. Our findings suggest that serum calcium might be considered as an additional component of the MSy.

In addition, serum calcium was associated with a vast array of NCCM risk factors, including proxies for insulin resistance and markers of oxidative stress, independently of the conventional MSy risk factors. Although the associations with BP [1], insulin resistance [24] and dyslipidemia [3] have been previously described, we are not aware that this was reported for serum uric acid, homocysteine, or GGT levels in a large-scale population-based sample. The associations of calcium levels and uric acid have been reported in patients with primary hyperparathyroidism [25], [26].

We reported that fasting insulin was significantly associated with serum calcium, independently of conventional cardio-metabolic risk factors. Our results were consistent with those of large-scale studies that found elevated serum calcium levels to be associated with insulin resistance, measured using fasting insulin or homeostatic model assessment [2], [27], [28]. By contrast, serum calcium has not been shown to be associated with insulin secretion [24], [27]. In our study, the negative association of serum adiponectin with serum calcium is concordant with the hypothesis that serum calcium is a marker of insulin resistance because adiponectin levels are decreased in patients with type 2 diabetes [29] and in insulin resistance states [30].

Homocysteine, a sulfur-containing amino acid resulting from the metabolism of methionine and a marker of animal protein consumption, has been previously associated with the MSy in some studies [10], but not with the components of the MSy in healthy men [31]. Homocysteine is an independent risk factor for cardiovascular disease, most probably through its role on atherogenesis and oxidative stress [32]. No relationship between homocysteine and Ca_c_ has been described so far, although several studies pointed out the role of homocysteine and oxidative stress on bone and thus, the indirect role of homocysteine on calcium metabolism [33], [34].

In humans, uric acid represents the final catabolic pathway for purines. Uricase activity has been lost over evolution in higher primates; consequently, humans have high uric acid plasma levels. This might confer an evolutionary advantage, maybe through its effect on oxidation. However, the role of uric acid has on oxidative metabolism is ambiguous. On one hand, it is a powerful anti-oxidant, representing about half of the anti-oxidant scavenging capacities of the plasma. On the other hand, its intracellular metabolism generates superoxide anions and other reactive oxygen species [35]. A positive association of uric acid and Ca_c_ levels has not been reported before, even though each of them have been associated with the MSy.[9] A small study has shown a negative association between calcemia, uric acid levels and xanthine oxidase activity in 2 healthy volonteers [36]. The association between Ca_c_ and uric acid levels lifts up new hypothesis about links between the metabolisms of uric acid and calcium. Both electrolytes are mainly reabsorbed in the proximal tubule of the kidney. They are dependent on sodium reabsorption, and on the renin-angiotensin-aldosterone system. The calcium ATP-ase (PMCA) [37] and the sodium/calcium exchanger (NCX1) [38] have been shown to be regulated by reactive oxygen species and are also involved in calcium reabsorption by the kidney [39]. Overall, the association of Ca_c_ with the NCCM risk factor uric acid raises new hypothesis which should be explored through new further studies.

GGT is an enzyme found mainly in the liver. It is involved in glutathione metabolism, amino acid transfers, and leukotriene metabolism [40]. An association between a progressive increase of GGT activity and the number of the MSy components has recently been reported [11].

GGT is also a marker of oxidative stress, even within its normal range [41]–[45]. Apart from the typical serum GGT elevation due to oxidative stress in the liver after alcohol consumption, GGT could be an early marker of oxidative stress related to atherosclerosis, diabetes and other cardiovascular disease.

In our study, uric acid, homocysteine and GGT were NCCM risk factors most strongly associated with serum Ca_c._ To the best of our knowledge, this is the first time that associations of serum calcium with uric acid, homocysteine or GGT are studied and positive associations reported. Oxidative stress and calcium have been extensively studied in the context of calcium transport and signaling [46]. At the cellular level, oxidative stress causes calcium influx into the cytoplasm, mitochondria, and nuclei. The association between serum Ca_c_ and oxidative stress related factors needs to be further explored. Of note, HDL-cholesterol, a lipoprotein with anti-oxidant proprieties, was not significantly associated with Ca_c_. In a previous population-based study [5], Ca_c_ was associated with HDL-cholesterol but only after excluding individuals with mild primary hyperparathyroidism . Because parathyroid hormone (PTH) was not available in our study we cannot exclude a similar association.

We reported higher CRP levels in the upper Ca_c_ quintiles, thought not significant. Higher CRP levels were associated with higher calcium levels in chronic hemodialysis patients [47]. General population-based studies on the association of calcium levels and CRP are lacking. The strong associations of Ca_c_ and uric acid, homocysteine and GGT, and the lack of association with CRP suggest that Ca_c_ is particularly related to oxidative stress rather than to inflammation.

One should differentiate the issue of calcium intake and the one of tightly regulated serum calcium levels. We found serum calcium to be associated with a wide-range of metabolic disturbances. Our findings provide no information on whether or not dietary calcium intake plays a role in these metabolic disturbances.

### Limitations

Our study has limitations. First, neither vitamin D nor PTH or other new regulator of mineral metabolism (e.g., FGF-23, Klotho) are available in our sample and we cannot explore whether the association of serum calcium with these metabolic disturbances is the result of, or mediated by, vitamin D or PTH action. Second, we assigned the same weight to each cardio-metabolic risk factor to derive the NCCM score. While we could have used unequal weights, an equal weight approach has been privileged to be concordant with the ATP III MSy criteria. Third, participants included in the analysis differed by important characteristics from the entire cohort, and thus our conclusions may not be representative of the general Lausanne population. Finally, the results from a cross-sectional study do not allow inferring causality.

While considering these limitations, our results confirmed previous associations between serum calcium and MSy and suggested that calcium metabolism could be considered as an additional feature of the MSy. In addition, calcium metabolism was associated with NCCM risk factors, including markers of insulin resistance, independently of the conventional components of the MSy and of BMI. The novel associations with oxidative stress markers were the strongest and deserve further explorations.

## Supporting Information

Table S1 **Clinical chemistry and biological makers measured in the CoLaus study with analytical procedures, maximum inter and intra-batch coefficient of variation and manufacturer.** Adapted from Firman et al.
(DOCX)

Table S2 **Comparison of variable in participants excluded because of missing measurements.**
(DOCX)

Table S3 **Adjusted conventional and non-conventional metabolic syndrome components, by sex-specific albumin-corrected calcium quintiles. (Men, N = 1,976).** *SBP = systolic blood pressure; DBP = diastolic blood pressure; uCRP = ultrasensitive C reactive protein. *Results are medians [95% confidence intervals] adjusted for age, smoking, alcohol consumption, menopause status, eGFR, and thiazide use. **Robust regression was used to model HDL-cholesterol.
(DOCX)

Table S4 **Adjusted conventional and non-conventional metabolic syndrome components, by sex-specific albumin-corrected calcium quintiles. (Women, N = 2,255).** *SBP =  systolic blood pressure; DBP =  diastolic blood pressure; uCRP = ultrasensitive C reactive protein. *Results are median [95% confidence intervals] adjusted for age, smoking, alcohol consumption, menopause status, eGFR, and thiazide use. **Robust regression was used to model HDL-cholesterol.
(DOCX)

Table S5 **Adjusted standardized associations between cardio-metabolic risk factors and albumin-corrected calcium.** *p value<0.001, **p value<0.05. Standard deviation = 1 for all standardized coefficients. All variables adjusted for age, smoking, alcohol consumption, menopause status, eGFR, and thiazide use. Non-conventional cardio-metabolic risk factors are additionally adjusted for conventional cardio-metabolic risk factors.
(DOCX)

Table S6 **Albumin-corrected calcium, by number of metabolic syndrome components.** *adjusted for sex (if appropriate), age, smoking, alcohol consumption, menopause status (if appropriate), eGFR, and thiazide use.
(DOCX)

Table S7 **Albumin-corrected calcium, by number of non-conventional cardio-metabolic risk factors.** *adjusted for sex (if appropriate), age, smoking, alcohol consumption, menopause status (if appropriate), eGFR, thiazide use, and MSy components
(DOCX)
