# Supplementary material for: Serum Calcium Levels Are Associated with Novel Cardiometabolic Risk Factors in the Population-Based CoLaus Study
Source: PLoS One. 2011 Apr 21;6(4):e18865. doi: 10.1371/journal.pone.0018865 (PMC3080882; doi:10.1371/journal.pone.0018865)
Supplement: Table S1 — Clinical chemistry and biological makers measured in the CoLaus study with analytical procedures, maximum inter and intra-batch coefficient of variation and manufacturer. Adapted from Firman et al. (DOCX) [file pone.0018865.s001.docx]

**Supplementary Table S1** Clinical chemistry and biological makers measured in the CoLaus study with analytical procedures, maximum inter and intra-batch coefficient of variation and manufacturer. Adapted from Firman et al.

| **Marker** | **Type of assay** | **Maximum inter and intra-batch CVs** | **Manufacturer** |
| --- | --- | --- | --- |
| Adiponectin | ELISA | 8.3% - 8.3% | R&D Systems, Inc, Minneapolis, USA |
| Albumin | Bromocresol green | 2.5% – 0.4% | Roche Diagnostics, CH |
| Apolipopoprotein B (ApoB) | Turbidimetry | 8.7% - 7.6% | Polymedco, Chicago, USA |
| Calcium | O-cresolphtalein | 2.1% – 1.5% | Roche Diagnostics, CH |
| Creatinine (serum and urine) | Jaffe kinetic compensated method | 2.9% – 0.7% | Roche Diagnostics, CH |
| Gamma glutamyl transferase (GGT) | Optimized standard method according to the IFCC, at 37°C | 1.6% – 0.4% | Roche Diagnostics, CH |
| Glucose | Glucose dehydrogenase | 2.1% – 1.0% | Roche Diagnostics, CH |
| HDL-cholesterol | CHOD-PAP + PEG + cyclodextrin | 3.6% – 0.9% | Roche Diagnostics, CH |
| ultrasensitive CRP (hsCRP) | Immunoassay and latex HS | 4.6% – 1.3% | Roche Diagnostics, CH |
| Homocystein | High pressure liquid chromatography following ammonium 7-fluorobenzo-2-oxa-1, 3-diazole -4-sulphonate (SBD-F) derivatisation | 3.1% – 2.9% | Agilent 1100 apparatus |
| Insulin | Solid-phase, two-site chemiluminescent immunometric assay | Maximum intra-assay CV of 13.7%. | Diagnostic Products Corporation, Los Angeles, USA |
| LDL particle size | Polyacrylamide gel electrophoresis, Lipoprint LDL kit^®^, | 1.5%-0.5% | Quantimetrix Corporation, CA, USA |
| Leptin | ELISA | 12.8% - 5.8% | American Laboratory Products Company, Windham, USA |
| Triglycerides | GPO-PAP | 2.9%-1.5% | Roche Diagnostics, CH |
| Uric acid | uricase-PAP | 1.0% – 0.5% | Roche Diagnostics, CH |
