# Supplementary material for: Serum Calcium Levels Are Associated with Novel Cardiometabolic Risk Factors in the Population-Based CoLaus Study
Source: PLoS One. 2011 Apr 21;6(4):e18865. doi: 10.1371/journal.pone.0018865 (PMC3080882; doi:10.1371/journal.pone.0018865)
Supplement: Table S2 — Comparison of variable in participants excluded because of missing measurements. (DOCX) [file pone.0018865.s002.docx]

**Supplementary Table S2 Comparison of Variable in Participants Excluded Because of Missing Measurements**

|  | **Included in Analyses**  **(N=4,231)** | | **Excluded from Analysis**  **(N=1,957)** | |  |
| --- | --- | --- | --- | --- | --- |
|  | **Mean or %** | **SD** | **Mean or %** | **SD** | **P value** |
| Age (years) | 53.6 | 10.9 | 51.8 | 10.4 | **<0.001** |
| Body mass index (kg/m^2^) | 26.1 | 4.2 | 25.3 | 5.2 | **<0.001** |
| Cigarette smoking (%) | 27.2 | - | 30.6 | - | **<0.001** |
| Regular alcohol consumption (%) | 26.3 | - | 24.9 | - | 0.241 |
| Post-menopause (%) | 30.4 | - | 27.4 | - | **0.017** |
| Hypertension (%) | 38.1 | - | 31.4 | - | **<0.001** |
| Diabetes (%) | 6.6 | - | 6.2 | - | 0.518 |
| eGFR (ml/min/1.73m^2^) | 83.1 | 16.3 | 84.6 | 17.0 | **0.011** |
| Serum calcium (mmol/L) | 2.29 | 0.09 | 2.28 | 0.10 | **0.003** |
| Serum albumin (g/L) | 44.2 | 2.5 | 44.3 | 2.6 | 0.231 |
| Albumin-corrected calcium (mmol/L) | 2.20 | 0.08 | 2.21 | 0.09 | **<0.001** |
