# Supplementary material for: Serum Calcium Levels Are Associated with Novel Cardiometabolic Risk Factors in the Population-Based CoLaus Study
Source: PLoS One. 2011 Apr 21;6(4):e18865. doi: 10.1371/journal.pone.0018865 (PMC3080882; doi:10.1371/journal.pone.0018865)
Supplement: Table S3 — Adjusted conventional and non-conventional metabolic syndrome components, by sex-specific albumin-corrected calcium quintiles. (Men, N = 1,976). *SBP = systolic blood pressure; DBP = diastolic blood pressure; uCRP = ultrasensitive C reactive protein. *Results are medians [95% confidence intervals] adjusted for age, smoking, alcohol consumption, menopause status, eGFR, and thiazide use. **Robust regression was used to model HDL-cholesterol. (DOCX) [file pone.0018865.s003.docx]

**Supplementary Table S3.** Adjusted conventional and non-conventional metabolic syndrome components, by sex-specific albumin-corrected calcium quintiles. (Men, N=1,976).*

|  | **Q1**  **[2.10 mmol/L]**  **(N=377)** | **Q2**  **[2.17 mmol/L]**  **(N=399)** | **Q3**  **[2.21 mmol/L]**  **(N=451)** | **Q4**  **[2.25 mmol/L]**  **(N=380)** | **Q5**  **[2.32 mmol/L]**  **(N=369)** | **P value**  **for trend** |
| --- | --- | --- | --- | --- | --- | --- |
| **CONVENTIONAL** |  |  |  |  |  |  |
| SBP (mm Hg) | 130 | 131 | 132 | 133 | 135 | **<0.001** |
|  | [128-131] | [130-132] | [131-133] | [132-135] | [133-136] |  |
| DBP (mm Hg) | 79.8 | 80.6 | 81.3 | 82.0 | 82.8 | **<0.001** |
|  | [78.8-80.8] | [79.9-81.3] | [80.7-81.9] | [81.3-82.7] | [81.8-83.8] |  |
| HDL-cholesterol (mmol/L)** | 1.37 | 1.38 | 1.38 | 1.39 | 1.40 | 0.195 |
|  | [1.34-1.39] | [1.36-1.39] | [1.37-1.40] | [1.37-1.40] | [1.37-1.42] |  |
| Triglycerides (mmol/L) | 1.24 | 1.29 | 1.35 | 1.40 | 1.45 | **0.001** |
|  | [1.17-1.31] | [1.24-1.34] | [1.30-1.39] | [1.35-1.45] | [1.38-1.53] |  |
| Fasting blood glucose (mmol/L) | 5.60 | 5.61 | 5.62 | 5.63 | 5.64 | 0.410 |
|  | [5.54-5.65] | [5.57-5.65] | [5.58-5.65] | [5.59-5.67] | [5.58-5.69] |  |
| Waist circumference (cm) | 96.4 | 96.4 | 96.4 | 96.4 | 96.4 | 1.000 |
|  | [95.5-97.4] | [95.8-97.1] | [95.9-97.0] | [95.8-97.1] | [95.5-97.4] |  |
| **NON CONVENTIONAL** |  |  |  |  |  |  |
| ADIPOSITY |  |  |  |  |  |  |
| Fat mass (kg) | 19.2 | 19.4 | 19.5 | 19.7 | 19.9 | 0.159 |
|  | [18.6-19.8] | [18.9-19.8] | [19.2-19.9] | [19.3-20.1] | [19.3-20.5] |  |
| Leptin (ng/mL) | 6.98 | 6.79 | 6.60 | 6.41 | 6.21 | 0.066 |
|  | [6.49-7.47] | [6.45-7.13] | [6.32-6.88] | [6.06-6.75] | [5.72-6.71] |  |
| LIPID |  |  |  |  |  |  |
| LDL-cholesterol (mmol/L) | 3.23 | 3.32 | 3.42 | 3.51 | 3.61 | **<0.001** |
|  | [3.16-3.29] | [3.28-3.37] | [3.38-3.45] | [3.47-3.56] | [3.54-3.67] |  |
| LDL size (angstrom) | 272.0 | 271.8 | 271.6 | 271.4 | 271.1 | **0.02** |
|  | [271.6-272.4] | [271.5-272.1] | [271.3-271.8] | [271.0-271.7] | [270.7-271.6] |  |
| Apolipoprotein B (mg/dL) | 149 | 150 | 152 | 153 | 154 | 0.408 |
|  | [141-157] | [145-156] | [147-156] | [147-159] | [146-163] |  |
| INSULIN |  |  |  |  |  |  |
| Fasting insulin (μU/mL) | 7.48 | 7.85 | 8.22 | 8.58 | 8.95 | **0.002** |
|  | [6.92-8.05] | [7.45-8.24] | [7.90-8.54] | [8.18-8.98] | [8.38-9.52] |  |
| Adiponectin (μg/mL) | 6547 | 6352 | 6156 | 5961 | 5766 | **0.02** |
|  | [6155-6938] | [6078-6625] | [5934-6379] | [5684-6239] | [5370-6163] |  |
| INFLAMMATION |  |  |  |  |  |  |
| uCRP (mg/L) | 1.35 | 1.38 | 1.41 | 1.44 | 1.47 | 0.17 |
|  | [1.24-1.46] | [1.31-1.46] | [1.35-1.47] | [1.37-1.52] | [1.37-1.58] |  |
| OXYDATIVE STRESS |  |  |  |  |  |  |
|  |  |  |  |  |  |  |
| Serum uric acid (μmol/L) | 346 | 353 | 360 | 368 | 375 | **<0.001** |
|  | [340-352] | [349-356] | [357-364] | [363-372] | [368-381] |  |
| Homocystein (μmol/L) | 10.18 | 10.42 | 10.65 | 10.89 | 11.12 | **<0.001** |
|  | [9.94-10.42] | [10.25-10.58] | [10.52-10.79] | [10.72-11.05] | [10.88-11.36] |  |
| GGT (UI/L) | 26.72 | 28.78 | 30.85 | 32.91 | 34.97 | **<0.001** |
|  | [24.95-28.49] | [27.55-30.02] | [29.84-31.85] | [31.66-34.16] | [33.18-36.76] |  |

SBP= systolic blood pressure; DBP= diastolic blood pressure; uCRP= ultrasensitive C reactive protein

*Results are medians [95% confidence intervals] adjusted for age, smoking, alcohol consumption, menopause status, eGFR, and thiazide use. **Robust regression was used to model HDL-cholesterol.
