# Supplementary material for: Serum Calcium Levels Are Associated with Novel Cardiometabolic Risk Factors in the Population-Based CoLaus Study
Source: PLoS One. 2011 Apr 21;6(4):e18865. doi: 10.1371/journal.pone.0018865 (PMC3080882; doi:10.1371/journal.pone.0018865)
Supplement: Table S4 — Adjusted conventional and non-conventional metabolic syndrome components, by sex-specific albumin-corrected calcium quintiles. (Women, N = 2,255). *SBP = systolic blood pressure; DBP = diastolic blood pressure; uCRP = ultrasensitive C reactive protein. *Results are median [95% confidence intervals] adjusted for age, smoking, alcohol consumption, menopause status, eGFR, and thiazide use. **Robust regression was used to model HDL-cholesterol. (DOCX) [file pone.0018865.s004.docx]

**Supplementary Table S4.** Adjusted conventional and non-conventional metabolic syndrome components, by sex-specific albumin-corrected calcium quintiles. (Women, N=2,255).*

|  | **Q1**  **[2.11 mmol/L] (N=526)** | **Q2**  **[2.18 mmol/L]**  **(N=401)** | **Q3**  **[2.22 mmol/L]**  **(N=495)** | **Q4**  **[2.26 mmol/L]**  **(N=386)** | **Q5**  **[2.34 mmol/L]**  **(N=447)** | **P value for trend** |
| --- | --- | --- | --- | --- | --- | --- |
| **CONVENTIONAL** |  |  |  |  |  |  |
| SBP (mm Hg) | 121 | 122 | 124 | 125 | 126 | **<0.001** |
|  | [120-123] | [121-123] | [123-124] | [124-126] | [124-127] |  |
| DBP (mm Hg) | 76.1 | 76.6 | 77.2 | 77.7 | 78.2 | **<0.001** |
|  | [75.3-76.8] | [76.0-77.1] | [76.7-77.6] | [77.2-78.3] | [77.5-79.1] |  |
| HDL-cholesterol (mmol/L)** | 1.76 | 1.77 | 1.77 | 1.77 | 1.78 | 0.819 |
|  | [1.73-1.79] | [1.75-1.79] | [1.75-1.79] | [1.75-1.80] | [1.74-1.81] |  |
| Triglycerides (mmol/L) | 0.96 | 1.00 | 1.02 | 1.06 | 1.09 | **0.001** |
|  | [0.92-1.00] | [0.97-1.02] | [1.00-1.05] | [1.03-1.08] | [1.05-1.13] |  |
| Fasting blood glucose (mmol/L) | 5.19 | 5.21 | 5.23 | 5.25 | 5.26 | 0.059 |
|  | [5.16-5.23] | [5.19-5.24] | [5.21-5.25] | [5.22-5.27] | [5.22-5.30] |  |
| Waist circumference (cm) | 81.7 | 81.9 | 82.4 | 82.8 | 83.1 | **0.036** |
|  | [80.7-82.8] | [81.2-82.7] | [81.8-83.5] | [81.9-83.5] | [82.0-84.2] |  |
| **NON CONVENTIONAL** |  |  |  |  |  |  |
| ADIPOSITY |  |  |  |  |  |  |
| Fat mass (kg) | 21.5 | 21.8 | 22.2 | 22.5 | 22.9 | **0.002** |
|  | [20.9-22.2] | [21.3-22.2] | [21.8-22.6] | [22.1-23.0] | [22.2-23.5] |  |
| Leptin (ng/mL) | 14.95 | 14.95 | 14.94 | 14.94 | 14.93 | 0.972 |
|  | [13.9-15.9] | [14.2-15.6] | [14.3-15.5] | [14.1-15.6] | [13.8-16.0] |  |
| LIPID |  |  |  |  |  |  |
| LDL-cholesterol (mmol/L) | 3.11 | 3.17 | 3.24 | 3.30 | 3.36 | **<0.001** |
|  | [3.03-3.19] | [3.11-3.22] | [3.19-3.28] | [3.24-3.36] | [3.28-3.45] |  |
| LDL size (angstrom) | 273.7 | 273.7 | 273.6 | 273.5 | 273.5 | **0.018** |
|  | [273.5-273.9] | [273.6-273.8] | [273.5-273.7] | [273.4-273.7] | [273.3-273.7] |  |
| Apolipoprotein B (mg/dL) | 135 | 136 | 135 | 135 | 135 | 0.690 |
|  | [129-141] | [132-140] | [132-139] | [131-139] | [129-141] |  |
| INSULIN |  |  |  |  |  |  |
| Fasting insulin (μU/mL) | 5.93 | 6.22 | 6.48 | 6.75 | 7.02 | **<0.001** |
|  | [5.67-6.20] | [6.04-6.41] | [6.32-6.63] | [6.55-6.94] | [6.74-7.30] |  |
| Adiponectin (μg/mL) | 10872 | 10720 | 10413 | 10189 | 9955 | **0.006** |
|  | [10343-11402] | [10352-11088] | [10108-10718] | [9801-10578] | [9398-10512] |  |
| INFLAMMATION |  |  |  |  |  |  |
| uCRP (mg/L) | 1.47  [1.32-1.62] | 1.44  [1.34-1.55] | 1.44  [1.35-1.53] | 1.43  [1.32-1.53] | 1.41  [1.26-1.57] | 0.923 |
| OXYDATIVE STRESS |  |  |  |  |  |  |
| Serum uric acid (μmol/L) | 251 | 261 | 267 | 275 | 282 | **<0.001** |
|  | [246-257] | [258-265] | [264-269] | [271-278] | [277-288] |  |
| Homocystein (μmol/L) | 8.10 | 8.61 | 8.89 | 9.30 | 9.69 | **<0.001** |
|  | [7.92-8.28] | [8.48-8.73] | [8.79-9.00] | [9.17-9.43] | [9.50-9.88] |  |
| GGT (UI/L) | 15.2 | 15.9 | 16.6 | 17.3 | 18.1 | **<0.001** |
|  | [14.6-15.7] | [15.5-16.3] | [16.3-16.9] | [16.9-17.8] | [17.4-18.7] |  |

SBP= systolic blood pressure; DBP= diastolic blood pressure; uCRP= ultrasensitive C reactive protein

*Results are median [95% confidence intervals] adjusted for age, smoking, alcohol consumption, menopause status, eGFR, and thiazide use. **Robust regression was used to model HDL-cholesterol.
