# Supplementary material for: Serum Calcium Levels Are Associated with Novel Cardiometabolic Risk Factors in the Population-Based CoLaus Study
Source: PLoS One. 2011 Apr 21;6(4):e18865. doi: 10.1371/journal.pone.0018865 (PMC3080882; doi:10.1371/journal.pone.0018865)
Supplement: Table S5 — Adjusted standardized associations between cardio-metabolic risk factors and albumin-corrected calcium. *p value<0.001, **p value<0.05. Standard deviation = 1 for all standardized coefficients. All variables adjusted for age, smoking, alcohol consumption, menopause status, eGFR, and thiazide use. Non-conventional cardio-metabolic risk factors are additionally adjusted for conventional cardio-metabolic risk factors. (DOCX) [file pone.0018865.s005.docx]

**Supplementary Table S5 Adjusted standardized associations between cardio-metabolic risk factors and albumin-corrected calcium**

| **Cardio-metabolic**  **risk factor** | **Men+Women**  **(N=4,231)**  **Adjusted standardized beta coefficients** | **Men**  **(N=1,976)**  **Adjusted standardized beta coefficients** | **Women**  **(N=2,255)**  **Adjusted standardized beta coefficients** |
| --- | --- | --- | --- |
| ***Conventional*** | |  |  |
| Systolic blood pressure | 0.0320 | 0.0605 | 0.0001 |
| Diastolic blood pressure | 0.0285 | <0.0001 | 0.5328 |
| HDL cholesterol | 0.0019 | 0.0026 | 0.0006 |
| _log_Triglycerides | 0.0765* | 0.0763** | 0.0639** |
| Fasting glucose | 0.0366** | 0.0470** | 0.0277 |
| Waist circumference | 0.0038 | -0.0183 | 0.0197 |
| ***Non-conventional*** | |  |  |
| Fat mass | 0.0712** | 0.0293 | 0.0822** |
| LDL cholesterol | 0.0621* | 0.0576** | 0.0592** |
| _cubic_LDL particle size | -0.0418** | -0.0467 | -0.0382 |
| _log_Insulin | 0.0389** | 0.0414 | 0.0407 |
| _Log_Adiponectin | -0.0797* | -0.0557** | -0.0867* |
| Uric acid | 0.0887* | 0.0510** | 0.1015* |
| _log_Homocysteine | 0.0793* | 0.0722** | 0.0790* |
| GGT | 0.0832* | 0.1260* | 0.0378 |

*p value<0.001, **p value<0.05

Standard deviation=1 for all standardized coefficients.

All variables adjusted for age, smoking, alcohol consumption, menopause status, eGFR, and thiazide use.

Non-conventional cardio-metabolic risk factors are additionally adjusted for conventional cardio-metabolic risk factors.
