# Supplementary material for: Serum Calcium Levels Are Associated with Novel Cardiometabolic Risk Factors in the Population-Based CoLaus Study
Source: PLoS One. 2011 Apr 21;6(4):e18865. doi: 10.1371/journal.pone.0018865 (PMC3080882; doi:10.1371/journal.pone.0018865)
Supplement: Table S6 — Albumin-corrected calcium, by number of metabolic syndrome components. *adjusted for sex (if appropriate), age, smoking, alcohol consumption, menopause status (if appropriate), eGFR, and thiazide use. (DOCX) [file pone.0018865.s006.docx]

**Supplementary Table S6** **Albumin-corrected calcium, by number of metabolic syndrome components**

|  | **Men+Women**  **(N=4,231)** | | **Men**  **(N=1,976)** | | **Women**  **(N=2,255)** | |
| --- | --- | --- | --- | --- | --- | --- |
|  | **Adjusted* Predicted Ca_c_ (SD)** | | **Adjusted* Predicted Ca_c_ (SD)** | | **Adjusted* Predicted Ca_c_ (SD)** | |
| **Number of metabolic syndrome components** | **Not adjusted for BMI** | **Adjusted for BMI** | **Not adjusted for BMI** | **Adjusted for BMI** | **Not adjusted for BMI** | **Adjusted for BMI** |
| 0 | 2.204 (0.002) | 2.205 (0.003) | 2.200 (0.004) | 2.200 (0.005) | 2.209 (0.004) | 2.210 (0.004) |
| 1 | 2.210 (0.002) | 2.211 (0.002) | 2.205 (0.004) | 2.205 (0.004) | 2.216 (0.004) | 2.217 (0.004) |
| 2 | 2.218 (0.003) | 2.218 (0.003) | 2.213 (0.004) | 2.213 (0.004) | 2.220 (0.005) | 2.216 (0.005) |
| 3 | 2.229 (0.004) | 2.228 (0.004) | 2.220 (0.005) | 2.220 (0.006) | 2.233 (0.008) | 2.230 (0.008) |
| 4 | 2.226 (0.007) | 2.225 (0.007) | 2.220 (0.009) | 2.220 (0.010) | 2.224 (0.136) | 2.221 (0.013) |
| 5 | 2.261 (0.013) | 2.260 (0.014) | 2.248 (0.021) | 2.248 (0.022) | 2.260 (0.022) | 2.261 (0.021) |
| P value for non-linearity | 0.40 | 0.43 | 0.91 | 0.91 | 0.77 | 0.51 |
| ***P value for linear trend*** | ***<0.0001*** | ***<0.0001*** | ***<0.0001*** | ***<0.0001*** | ***<0.0001*** | ***<0.0001*** |

* adjusted for sex (if appropriate), age, smoking, alcohol consumption, menopause status (if appropriate), eGFR, and thiazide use.
