# Supplementary material for: Serum Calcium Levels Are Associated with Novel Cardiometabolic Risk Factors in the Population-Based CoLaus Study
Source: PLoS One. 2011 Apr 21;6(4):e18865. doi: 10.1371/journal.pone.0018865 (PMC3080882; doi:10.1371/journal.pone.0018865)
Supplement: Table S7 — Albumin-corrected calcium, by number of non-conventional cardio-metabolic risk factors. *adjusted for sex (if appropriate), age, smoking, alcohol consumption, menopause status (if appropriate), eGFR, thiazide use, and MSy components (DOCX) [file pone.0018865.s007.docx]

**Supplementary Table S7** **Albumin-corrected calcium, by number of non-conventional cardio-metabolic risk factors**

|  | **Men+Women**  **(N=4,231)** | | **Men**  **(N=1,976)** | | **Women**  **(N=2,255)** | |
| --- | --- | --- | --- | --- | --- | --- |
|  | **Adjusted* Predicted Ca_c_ (SD)** | | **Adjusted* Predicted Ca_c_ (SD)** | | **Adjusted* Predicted Ca_c_ (SD)** | |
| **Number of non-conventional components** | **Not adjusted for BMI** | **Adjusted for BMI** | **Not adjusted for BMI** | **Adjusted for BMI** | **Not adjusted for BMI** | **Adjusted for BMI** |
| 0 | 2.184 (0.005) | 2.185 (0.005) | 2.216 (0.013) | 2.216 (0.013) | 2.195 (0.005) | 2.197 (0.005) |
| 1 | 2.199 (0.003) | 2.201 (0.004) | 2.193 (0.007) | 2.195 (0.008) | 2.207 (0.004) | 2.207 (0.004) |
| 2 | 2.207 (0.003) | 2.207 (0.003) | 2.203 (0.005) | 2.204 (0.005) | 2.215 (0.004) | 2.215 (0.004) |
| 3 | 2.215 (0.004) | 2.215 (0.003) | 2.197 (0.005) | 2.198 (0.005) | 2.234 (0.005) | 2.235 (0.005) |
| 4 | 2.230 (0.005) | 2.230 (0.004) | 2.216 (0.005) | 2.216 (0.005) | 2.244 (0.007) | 2.243 (0.006) |
| 5 | 2.235 (0.006) | 2.236 (0.005) | 2.225 (0.05) | 2.226 (0.06) | 2.246 (0.009) | 2.244 (0.009) |
| 6 | 2.240 (0.006) | 2.240 (0.007) | 2.229 (0.007) | 2.231 (0.007) | 2.251 (0.016) | 2.251 (0.015) |
| 7+ | 2.250 (0.009) | 2.247 (0.010) | 2.231 (0.010) | 2.233 (0.010) | 2.283 (0.028) | 2.280 (0.026) |
| P value for non-linearity | 0.75 | 0.82 | 0.30 | 0.33 | 0.85 | 0.77 |
| ***P value for linear trend*** | ***<0.0001*** | ***<0.0001*** | ***<0.0001*** | ***<0.0001*** | ***<0.0001*** | ***<0.0001*** |

* adjusted for sex (if appropriate), age, smoking, alcohol consumption, menopause status (if appropriate), eGFR, thiazide use, and MSy components
